# Surgical site infections after elective craniotomy for brain tumor: a study on potential risk factors and related treatments

**DOI:** 10.1186/s41016-023-00336-1

**Published:** 2023-08-08

**Authors:** Yifan Lv, Xiang Mao, Yuxuan Deng, Lanbing Yu, Junsheng Chu, Shuyu Hao, Nan Ji

**Affiliations:** 1 Department of Neurosurgery, Beijing Tiantan Hospital, Capital Medical University, Beijing, 100070 China; 2 Department of Neurosurgery, The First Affiliated Hospital of Anhui Medical University, Hefei, 230022 Anhui China

**Keywords:** Brain tumor, Elective craniotomy, Neurosurgery, Surgical site infection, Risk factors, Infection control

## Abstract

**Background:**

Surgical site infection (SSI) is a common complication following craniotomy that increases morbidity, mortality, and medical expenses. The objectives of this study were to determine the relevant risk factors associated with SSI after elective craniotomy for brain tumor and analyse the treatments for SSI.

**Methods:**

A retrospective nested case‒control study was conducted using data from patients who underwent craniotomy for brain tumor resection at the Neurosurgical Oncology Department No. 6 of Beijing Tiantan Hospital, Capital Medical University, between January 2019 and December 2021. Risk factors for SSI were determined using multivariate logistic regression analysis. We analyzed microbiological and related treatment data for different SSI types.

**Results:**

Among 2061 patients who underwent craniotomy for brain tumor, 31 had SSI (1.50%)*.* In the multivariate logistic regression analysis, body mass index (BMI) and operative duration were identified as independent risk factors for SSI. The most common microorganism isolated from SSIs was Staphylococcus epidermidis (22.9%), and drug sensitivity results showed that gram-positive bacteria were sensitive to linezolid, vancomycin and tigecycline, whereas gram-negative bacteria were sensitive to meropenem, cefepime and ceftazidime. Six of the seven patients who underwent bone flap removal due to osteomyelitis were infected with gram-negative bacteria.

**Conclusions:**

BMI and operative duration were identified as independent risk factors for SSI. Diabetes mellitus, previous ratio therapy, type of incision, recurrence tumor and other risk factors were not found to be associated with the occurrence of SSI in this study.

**Supplementary Information:**

The online version contains supplementary material available at 10.1186/s41016-023-00336-1.

## Background

Surgical site infection (SSI) after elective craniotomy can result in permanent disability, prolonged hospitalization, or unscheduled readmissions, all of which increase health-care expenses [1]. In previous studies, the incidence of SSI after craniotomy ranges from 1.2 to 5.3% [1–16]. Henri et al. reported that SSI after first glioblastoma resection is a severe condition, significantly influencing patient survival [4]. In another study, infection, a surgical complication in neurosurgery, was the most common reason for readmission [17].

To date, only a few studies have particularly focused on the risk factors for SSI after elective craniotomies for brain tumor [18]. We believe it is important to focus on surgical site infections for elective craniotomy for brain tumor. First, the incisions for elective craniotomy are mostly clean incisions compared to cranial trauma surgery and transsphenoidal surgery. Second, compared to patients undergoing cerebrovascular and bypass surgery, brain tumor patients, especially those with malignant brain tumors, have a poorer prognosis based on the disease itself. Therefore, avoiding surgical site infection while pursuing prolonged patient survival is particularly important for elective craniotomy for brain tumor.

Previous studies on SSI after craniotomy have mostly been limited to risk factor analysis, and the identified risk factors include advanced age, diabetes mellitus, malnutrition, preoperative steroid use, preoperative radiation, obesity, the length of surgery, clean-contaminated and contaminated surgery, and a history of recent neurosurgical intervention [13, 19].

The objective of this study was to determine the factors that contribute to the development of SSI after elective craniotomies for brain tumor resection to provide evidence for SSI prevention.

## Methods

### Baseline characteristics

Patients who underwent craniotomy for brain tumor resection at the neurosurgical oncology department NO. 6, Beijing Tiantan Hospital, Capital Medical University, between January 2019 and December 2021 were retrospectively assessed for this study.

The same operation preparation protocol was followed for all the patients; hair was shaved the day before surgery and skin was prepared preoperatively (using antiseptic iodine and alcohol). Reference Global Guidelines For The Prevention Of Surgical Site Infections (2018) recommendations, while considering the half-life of the antibiotic [20]. All patients (if not allergic) received a single dose of the prophylactic antibiotic intravenous cefuroxime (1.5 g, with a half-life of approximately 80 min) at induction/before skin incision, repeated every 4 h during surgery. Antibiotics are not routinely administered after surgery unless the neurosurgeon suspects, intraoperatively, that the patient is at risk of infection (e.g., mastoid air cells or sinus opening, violation of sterility during surgery, coinfection before surgery).

### Definition and classification of SSIs

Researchers have debated the classification and definition of SSIs [4]. Based on the American Centers for Disease Control and Prevention (CDC) criteria for Surgical Site Infection (2017) [21], and in order to make the SSI false-positive rate as low as possible. We defined SSI as a purulent discharge requiring local debridement or osteomyelitis/abscess requiring reoperation, as well as positive cultures from secretion samples or CSF. This is comparable to the definition of SSI used by Sami et al. [12].

We divided SSI into the following three types: SSI-S (superficial infections involving only skin or subcutaneous tissue), SSI-D (deep infections including fascia and/or muscle), and SSI-OS (organ or space infections, such as osteomyelitis, meningitis, ventriculitis, or abscess), for more details on classification criteria, see the Additional Table S1. This is similar to the classification by Stienen et al. [7].

### Data collection

Basic data were collected using an electronic medical record system. Demographic data included sex, age, diabetes mellitus, BMI (body mass index: calculated from the height and weight provided in the admission record), previous radiotherapy (yes or no), recurrence (yes or no) and histological diagnoses. Furthermore, the following parameters were collected regarding the operations: consecutive operation (yes or no), type of incision, operative duration (measured from skin incision to last suture/closure), estimated blood loss, and length of postoperative hospital stay. With reference to a meta-analysis involving 26 studies [22] and the data we have collected above, we chose to examine BMI, diabetes mellitus, previous ratio therapy, recurrence tumor, type of incision, operative duration, and estimated blood loss as potential risk factors for SSI after elective craniotomy for brain tumor in this study. Finally, we collected results from microbiological cultures, drug sensitivity results, and the relevant treatment of SSI.

### Statistical analysis

Statistical analysis was performed using SPSS 26.0 and GraphPad Prism 8.3.0. A retrospective nested case‒control study was conducted to determine the possible risk factors for SSI. Each case of SSI was matched with two controls based on the following three criteria: age (± 0), sex, and period of procedure (± 12 months). For all variables, between-group comparisons were made using the *t* test/Wilcoxon rank-sum for continuous variables or the chi-square test/Fisher’s exact test for categorical variables as appropriate. Multivariate analysis using logistic regression to identify independent risk factors. ROC curves were drawn for independent prognostic factors. The best cut-off value was determined using Youden's Index, which maximizes the sum of sensitivity and specificity-1. The AUC of each independent prognostic factor was compared to determine its prognostic value. For descriptive analysis, microbiological cultures, drug sensitivity results and the relevant treatment of SSI were provided. Odds ratios (ORs) were obtained with corresponding 95% confidence intervals (CIs). Statistical significance was defined as *P* < 0.05.

## Results

### Prevalence of SSI and comparison of the baseline data

**Fig. 1:**
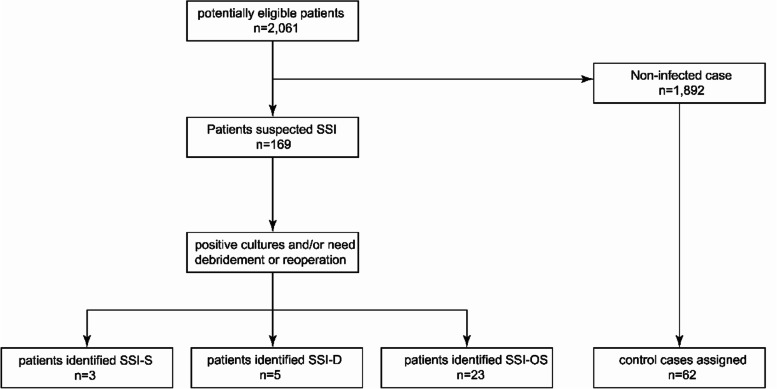
Summary of patient selection

A total of 2061 patients who underwent craniotomy for brain tumor resection between January 2019 and December 2021 met the inclusion criteria. Thirty-one cases of surgical site infections were identified, and 62 matched controls were selected based on three criteria (age, sex, period of procedure) from the remaining cases (Fig. 1). The overall infection rate was 1.50%, which was similar to the SSI rate after craniotomy for brain tumors reported by Laura G. Valentini et al. [16].

Table 1 shows a comparison of the basic data of the two groups. Significant differences in BMI (*P* = 0.007), duration of neurosurgery (*p* = 0.01), and length of postoperative hospital stay (*p* < 0.001) were identified in between-group comparisons.

**Table 1:** Comparison of baseline characteristics

Characteristic	Case(*n* = 31)	Controls(*n* = 62)	*P* value
Sex			1.0^a^
Male	20(64.5%)	40(64.5%)	
Female	11(35.5%)	22(35.5%)	
Age (years)			1.0^b^
Mean (SD)	45.9(15.1)	45.9(15.0)	
Median (IQR)	50(33–57)	50(33–57)	
Range	17–77	17–77	
BMI (kg/m^2^)			0.007^b^
Mean (SD)	26.0(4.8)	23.3(2.9)	
Median (IQR)	25.1(22.6–27.7)	23.4(21.4, 25.5)	
Range	18.7–41.0	15.6–28.6	
Diabetes mellitus (yes or no)	2(6.5%)	2(3.2%)	0.598^c^
Previous ratio-therapy (yes or no)	3(9.7%)	2(3.2%)	0.329^c^
Recurrence tumor (yes or no)	4(12.9%)	3(4.8%)	0.217^c^
Histological diagnosis			0.495^a^
High-grade glioma	14(45.2%)	17(27.4%)	
Low-grade glioma	4(12.9%)	11(17.7%)	
Meningiomas	6(19.4%)	17(27.4%)	
Hemangioblastoma	2(6.5%)	1(1.6%)	
Metastatic tumor	0(0%)	4(6.5%)	
Schwannomas	1(3.2%)	5(6.5%)	
Epidermoid cysts	1(3.2%)	2(3.2%)	
Other tumors	3(9.7%)	6(9.7%)	
Type of surgical wound^*^			0.745^a^
I (Clean)	28(90.3%)	53(85.5%)	
II (Clean/Contaminated)	3(9.7%)	9(14.5%)	
Operative duration(h)			0.001^b^
Mean(SD)	4.6(1.1)	3.8(1.0)	
Median (IQR)	4.5(4.0, 5.5)	3.9(3.0, 4.4)	
Range	2.8–6.8	1.9–7.0	
Estimated blood loss(ml)			0.137^b^
Mean(SD)	258.1(200.0)	211.29(101.4)	
Median (IQR)	200(100, 300)	200(150, 300)	
Range	100–1200	50–600	
Length of post-operative hospital stay (days)			< 0.001^b^
Mean(SD)	28.3(16.1)	9.7(2.9)	
Median (IQR)	23(20, 31)	9.0(7.8, 11.0)	
Range	14–94	6–20	

*Abbreviations*: *BMI* Body mass index, *SD* Standard deviation, *IQR* Interquartile range

^*^According to the classification criteria for surgical wound created by the Center for Disease Control and Prevention (I, clean; II, clean/contaminated; III, contaminated; and IV, dirty)

^a^chi-square test

^b^*t* test

^c^Fisher’s exact

### Risk factors and their predictive value

To determine relevant risk factors associated with SSI, BMI, diabetes mellitus, previous ratio therapy, recurrence tumor, type of incision, operative duration, and estimated blood loss were included in the univariate logistic regression analysis. The results showed that BMI and operative duration were associated with a higher risk of SSI. BMI (OR 1.24, 95% CI 1.06–1.44, *p* = 0.006) and operative duration (OR 2.09, 95% CI 1.31–3.34, *p* = 0.002) were also independent risk factors for post-craniotomy SSI according to the multivariate logistic regression analysis (Table 2).

**Table 2:** Multivariate analysis for the risk for surgical site infection

Covariate	*B*	Wals	*p* value	OR (95% CI)
BMI	0.21	7.52	0.006	1.24(1.06–1.44)
Operative duration	0.74	9.48	**0.002**	2.09(1.31–3.34)
Constant	− 9.05	15.26	< 0.001	< 0.001

*CI* Confidence interval, *OR* Odds ratio, *BMI* Body mass index

**Fig. 2:**
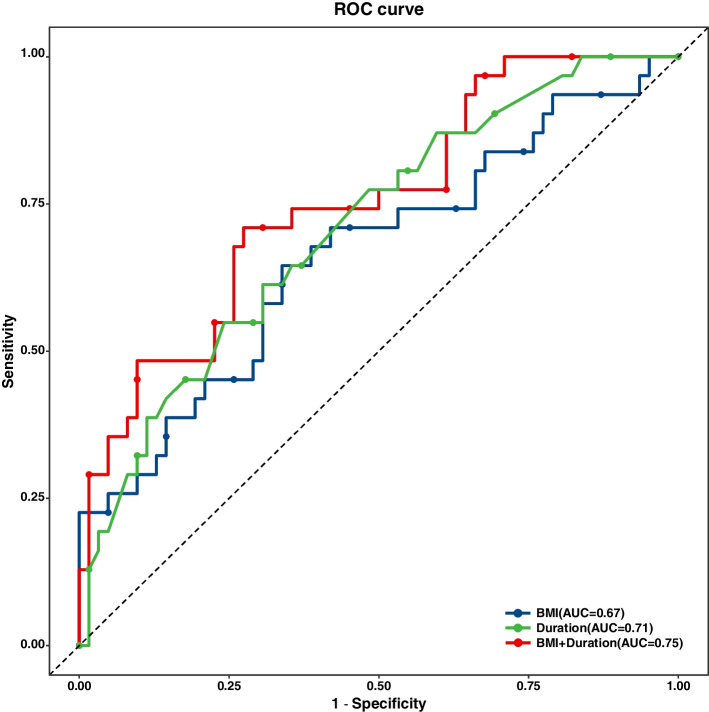
The ROC curve of BMI, operative duration, and the combination of BMI and operative duration for predictive evaluation for SSI

Logistic regression analysis was performed to obtain regression coefficients for independent risk factors, and then ROC analysis of BMI, operative duration, and the combination of the two factors was performed for the predictive evaluation of SSI. The optimal cut-off values of BMI, operative duration, and the combination of the two factors were 24.36 kg/m^2^, 4.465 h and 30.93, respectively. The AUCs were 0.67, 0.71 and 0.75, respectively. The sensitivity was 64.52%, 54.84%, and 72.58%, respectively. The specificity was 66.13%, 75.81% and 70.97%, respectively (Fig. 2 and Additional Table S2).

### Pathogenic microorganism results

**Fig. 3:**
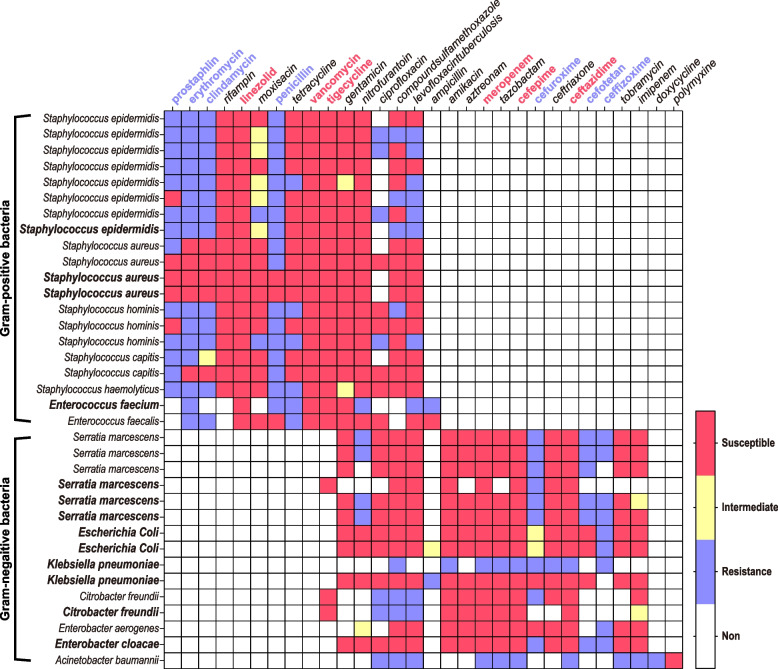
The Heatmap of drug sensitivity. In the column labels, bolded font indicates secretion pathogenic bacterial culture and regular font indicates a CSF bacterial culture. In the row labels, red font indicates relatively sensitive antibiotics and blue font represents relatively insensitive antibiotics

A total of 35 strains of nonrepetitive pathogenic bacteria were collected from the CSF and pyogenic samples, of which 57.1% (20 strains) were gram-positive bacteria and 42.9% (15 strains) were gram-negative bacteria. The most common pathogenic bacteria isolated from SSIs were *S. epidermidis* (22.9%), followed by *Serratia marcescens* (17.1%) and *Staphylococcus aureus* (11.4%). The drug sensitivity results showed that all gram-positive bacteria were sensitive to linezolid, vancomycin, and tigecycline, but most were insensitive to prostaphlin, erythromycin, clindamycin, and penicillin. Except for one strain of Klebsiella and *A. baumannii*, all gram-negative bacteria were sensitive to meropenem, cefepime and ceftazidime, but most were insensitive to cefuroxime, cefotetan and ceftizoxime (Table 3 and Fig. 3).

**Table 3:** Pathogens causing SSI after elective craniotomy for brain tumor

	Specimens resource		
Pathogenic bacteria	CSF	Pyogenic fluids	Quantity
Gram-positive bacteria			20 (57.1%)
Staphylococcus epidermidis	7	1	8 (22.9%)
Staphylococcus aureus	2	2	4 (11.4%)
Staphylococcus hominis	3	0	3 (8.6%)
Staphylococcus capitis	2	0	2 (5.7%)
Staphylococcus hemolyticus	1	0	1 (2.9%)
Enterococcus faecium	0	1	1 (2.9%)
Enterococcus faecalis	1	0	1 (2.9%)
Gram-negative bacteria			15 (42.9%)
Serratia marcescens	3	3	6 (17.1%)
Escherichia Coli	0	2	2 (5.7%)
Klebsiella pneumoniae	0	2	2 (5.7%)
Citrobacter freundii	1	1	2 (5.7%)
Acinetobacter baumannii	1	0	1 (2.9%)
Enterobacter aerogenes	1	0	1 (2.9%)
Enterobacter cloacae	0	1	1 (2.9%)
Total	22	13	35 (100%)

### Treatment and prognosis of SSI

**Fig. 4:**
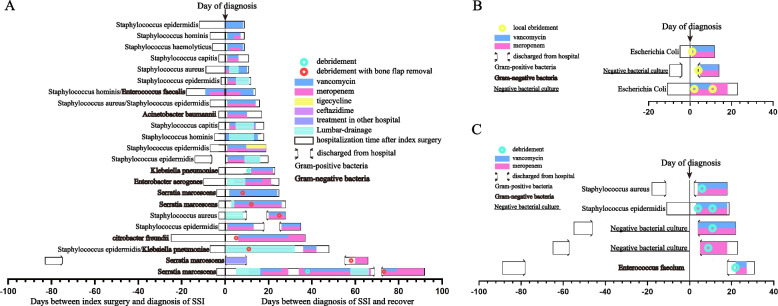
swimming plot. Detailed treatment procedures for different subtypes of SSI. **A** SSI-OS treatment procedures. **B** SSI-S treatment procedures. **C** SSI-D treatment procedures

We reviewed and summarized the treatment process of 31 cases of SSI that occurred after elective craniotomy for brain tumors. The treatment process mainly includes antibiotic treatment, lumbar drainage, local anaesthesia for debridement, general anaesthesia for thorough debridement, and bone flap removal due to osteomyelitis. The mean times from operation to SSI-S, SSI-D and SSI-OS were 8.7 days, 47.6 days, and 10.5 days, respectively. The mean times to SSI cure were 16.3 days, 22.6 days, and 25.7 days, respectively. The treatment required for each type of SSI was different. Patients with only positive CSF cultures in SSI-OS require only antibiotics and lumbar drainage. SSI-S requires local anaesthesia for debridement, and SSI-D and brain abscesses in SSI-OS require general anaesthesia for thorough debridement, whereas patients with osteomyelitis even need removal of bone flaps. In addition, it is noteworthy that four of the five SSI-D cases were found to be infected after discharge, and six of the seven patients who had bone flap removal due to osteomyelitis were infected with gram-negative bacteria (Fig. 4 and Additional Table S3).

## Discussion

### Incidence and classification of SSI

In numerous studies, the rates of SSI after neurosurgery varied due to differences in patient characteristics, operation indication, follow-up time, and SSI criteria [12]. For example, Stienen et al. defined SSI according to CDC standards [7], while Hsiu-Yin et al. defined SSI according to the National Healthcare Safety Network (NHSN) definition of SSI [9]. Consequently, comparing SSI incidence across studies with different criteria and demographics should be done with caution. Defining a universal diagnostic criterion for SSI after craniotomy is both worthwhile and urgent.

Proper classification of SSI is critical because deep or organ/space infections are associated with significantly increased disability and mortality compared with superficial infections. SSI-OS (organ/space infections) accounted for most SSI cases (74.2%) in the current investigation, which is consistent with research by Chiang et al. [9] but different from that reported by Buang et al. [23].

### BMI and operative duration

This study shows that the combination of BMI and operative duration has some predictive value but does not yet achieve high accuracy (the AUC of the combination of BMI and duration was 0.75 < 0.90). BMI was identified as an independent risk factor after craniotomy for brain tumor resection in this study. Connor et al. also identified BMI as a significant risk factor for the occurrence of SSI [13]. Adipose tissue has been reported to be insufficiently vascularized, which can make wound healing difficult. Furthermore, macro- and micronutrient deficiencies, which are common in overweight people, can compromise immune system function, thus increasing their risk for SSI [24]. This suggests that during the perioperative period, we should pay attention to overweight patients, as they may be at high risk for SSI.

The operative duration was found to be a significant independent risk factor for neurosurgery in numerous studies [5, 13, 16]. In this study, the operative duration was also found to be a significant independent risk factor for SSI. In a retrospective study of patients who had a craniotomy due to a brain tumor, Hardy et al. discovered that each additional hour spent in surgery resulted in a 43% increase in the odds ratio of developing an SSI [5]. Multiple factors influence the operative duration [16], and the presence of surgical trainees, surgical method and approach, surgeon proficiency, communication and logistics among operative specialists may also be important [1]. More bacteria could enter the incision by several routes, including airborne dissemination, surgical tools, and the patient's skin, as the duration of the operation lengthens [1]. Therefore, neurosurgeons should devise an ideal surgical strategy to efficiently reduce preoperative time.

However, diabetes mellitus, previous ratio therapy, type of incision, recurrence tumor and other risk factors reported in previous studies [19, 25, 26] were not found to be associated with the occurrence of SSI in this study. It is worth noting that diabetes mellitus is a significant predictor in most studies of surgical site infections. Unexpectedly, the results of this study showed that diabetes mellitus was not a risk factor for surgical site infection. We believe this is mainly due to the fact that we have avoided some infections by closely monitoring and strictly controlling the patient's blood glucose in the perioperative period. In addition, the relatively small size of the case group also influenced our findings.

### Pathogens causing SSI

Our statistics show that Staphylococcus species were the predominant microorganisms found in surgical site infections, similar to previous studies [6]. This demonstrated that the skin flora is the major source of infection. The results of our study suggest that single-shot antibiotic prophylaxis before surgery and routine dressing change after surgery at an appropriate frequency are of great importance.

It should be noted that although gram-negative bacteria are less common than gram-positive bacteria, they cause heavier infections. For example, six of the seven cases of osteomyelitis were infected by Gram-negative bacteria, suggesting a predisposition to cranial osteomyelitis when Gram-negative bacteria are infected. Therefore, to prevent the occurrence of osteomyelitis, radical treatment with early application of high-level antibiotics or thorough debridement should be undertaken when Gram-negative infection is suspected or confirmed.

In addition, analysis of the tumor composition of SSI patients revealed that there was no metastatic tumor in SSI cases. A recent study by Aikun Fu et al. found that intracellular bacteria in breast cancer tissues play a key role in the process of tumor metastasis [27], which suggests that intracellular bacteria no longer have a propensity for infection, but based on the small sample size of this study, evidence supporting this view is limited.

### Treatment of SSI

Due to additional treatment for SSI, the length of postoperative hospital stay and readmission rates can increase significantly. In this study, the mean length of postoperative hospital stay (28.3 ± 16.1) of patients with SSI was significantly longer than that of the control group (9.7 ± 2.9), and the maximum length of hospitalization for SSI treatment was 92 days. Ten out of 31 patients were readmitted for SSI treatment. Seven SSI patients underwent bone flap removal due to osteomyelitis, and some of them later underwent costly cranioplasty surgery. All these findings illustrate the high *health care* cost of SSI and the decreased well-being of the patient. It is therefore particularly crucial to identify patients at high risk of SSI for prevention*.*

In addition, it is important to know that the different subtypes are not independent of each other; SSI-S (superficial infection) may progress to SSI-D (deep infection), and deep infection may continue to progress to osteomyelitis or abscess in SSI-OS, so early diagnosis and treatment are extremely important. Based on the drug sensitivity results, it is certain that vancomycin combined with meropenem can treat almost all SSI-associated pathogens, and when vancomycin or meropenem allergies exist, we can use tigecycline and ceftazidime to replace these medications, respectively.

## Limitation

There are a few limitations in our study. First, only patients requiring local debridement or reoperation due to suspected infection or those with positive bacterial culture results were examined. Consequently, superficial infections that heal spontaneously or require only simple treatment in outpatient clinics are procedurally neglected. This means that the rate of SSI in our study was lower than the actual value, mainly because of the losses from SSI-S. Second, there were 45.2% of high-grade gliomas in the infected cases compared to 27.4% of controls, which may be due to reasons such as post-operative radiotherapy or their own poorer status. But based on the clinical data that could be collected so far, we do not have sufficient evidence to explain why there were more glioma patients in the infected cases, which requires further clinical research. Thirdly, this study was a single canter study and focused on surgical site infections following elective craniotomy for brain tumor. Therefore, the sample size is limited and the results are only applicable to elective craniotomy. Future multicenter studies on surgical site infections for brain tumor after elective craniotomy are expected to be conducted.

## Conclusions

There are few reports on the risk factors for SSI after elective craniotomy for brain tumor resection. This study provides some basis for neurosurgeons to prospectively identify high-risk groups and to better understand the required treatment for SSI.

In the current study, BMI and operative duration were identified as independent risk factors for SSI, and the combination of BMI and operative duration has some predictive value for SSI. The most common pathogenic bacteria isolated from SSIs were S. epidermidis, followed by S. marcescens and S. aureus. Gram-negative bacterial infections in SSI-OS are prone to complicate osteomyelitis. The drug sensitivity results show that empirical application of vancomycin combined with meropenem can treat almost all SSI-associated pathogens. The treatment required for each type of SSI is different, and early diagnosis and treatment are extremely important.

### Supplementary Information

**Additional file 1: Additional Table S1.** Surgical site infection criteria. **Additional Table S2.** Predictive value of BMI, duration of surgery and the combination of them for SSI. **Additional Table S3.** Treatment of Surgical site infection.

## Data Availability

The datasets used and/or analyzed during the current study are available from the corresponding author on reasonable request.

## Abbreviations

SSI: Surgical site infections
SSI-S: Superficial infections of SSI
SSI-D: Deep infections of SSI
SSI-OS: Organ or space infections of SSI
BMI: Body mass index
CSF: Cerebro-spinal fluid
SD: Standard deviation
IQR: Interquartile range
CI: Confidence interval
OR: Odds ratio
