# Supplementary material for: Surgical site infections after elective craniotomy for brain tumor: a study on potential risk factors and related treatments
Source: Chin Neurosurg J. 2023 Aug 8;9:23. doi: 10.1186/s41016-023-00336-1 (PMC10408142; doi:10.1186/s41016-023-00336-1)
Supplement: Supplementary file 1 — Additional file 1: Additional Table S1. Surgical site infection criteria. Additional Table S2. Predictive value of BMI, duration of surgery and the combination of them for SSI. Additional Table S3. Treatment of Surgical site infection. [file 41016_2023_336_MOESM1_ESM.pdf]

Additional Table 1 Surgical Site Infection Criteria

<table>
<tr><th>SSI Type</th><th>Time to event（days）</th><th>Depth of infection</th><th>patient has at least one of the following:</th><th>Required treatment</th></tr>
<tr><td rowspan="2">SSI-S</td><td rowspan="2">＜30</td><td rowspan="2">Skin or subcutaneous tissue of the incision</td><td>Purulent drainage</td><td rowspan="2">local anesthesia for debridement</td></tr>
<tr><td>Positive culture</td></tr>
<tr><td rowspan="2">SSI-D</td><td rowspan="2">＜90</td><td rowspan="2">deep soft tissues，fascia and/or muscle of the incision</td><td>Purulent drainage</td><td rowspan="2">general anesthesia for thorough debridement</td></tr>
<tr><td>Positive culture</td></tr>
<tr><td rowspan="4">SSI-OS</td><td rowspan="4">＜90</td><td rowspan="4">organ or space infections, such as osteomyelitis, meningitis, ventriculitis, abscess</td><td rowspan="2">Positive culture</td><td>Antibiotic Treatment</td></tr>
<tr><td>Lumbar-drainage</td></tr>
<tr><td rowspan="2">Images suggesting infection</td><td>general anesthesia for thorough debridement</td></tr>
<tr><td>bone flap removal</td></tr>
</table>

Additional Table 2 Predictive value of BMI, duration of surgery and the combination of them for SSI

	**Optimum Cut-Off value**	**AUC**	**95% CI**	**Sensitivity (%)**	**Specificity (%)**
**BMI**	24.36	0.67	0.54-0.79	64.52	66.13
**Duration of Surgery**	4.465	0.71	0.6-0.82	54.84	75.81
**combination**	30.93	0.75	0.65-0.86	72.58	70.97

Additional Table 3 Treatment of Surgical site infection

	SSI-S	SSI-D	SSI-OS
**Case**	3	5	23
**antibiotic treatment**	-	-	9
**antibiotic treatment +Lumbar-drainage**	-	-	6
**local anesthesia for debridement**	3	-	-
**general anesthesia for thorough debridement**	-	5	1
**bone flap removal due to osteomyelitis**	-	-	7
**Readmission due to SSI**	1	4	5
**Date of event for infection occurs**			
Mean	8.7	47.6	10.5
Median	10	55	6
Range	5-11	11-89	2-83
**Day for SSI treatment**			
Mean	16.3	22.6	25.7
Median	14	22	19
Range	12-23	18-31	9-92
